# Longitudinal Correlates of Suicide Ideation in People who use Drugs during the COVID-19 Pandemic

**DOI:** 10.21203/rs.3.rs-1910465/v1

**Published:** 2022-08-26

**Authors:** Kasey Claborn, Elizabeth Lippard, Fiona Conway

**Affiliations:** University of Texas at Austin; University of Texas at Austen; University of Texas at Austin

## Abstract

Some individuals may be more vulnerable to increased suicide-related thoughts and behavior in response to the COVID-19 pandemic but few studies have investigated risk factors that may be more predictive/specific to particular populations that are established to have a high risk for suicide, including gender differences in risk factors. We conducted a longitudinal study investigating risk factors for suicide ideation during the COVID-19 pandemic in adults who use alcohol and other drugs. Participants completed up to three surveys over a six-month follow-up period. Trait differences in substance use, COVID exposure/worry, trauma exposure, mood and post-traumatic stress disorder symptoms were investigated between individuals who presented with suicide ideation during course of study, compared to those that did not. Interactions with gender was investigated. State-related changes that relate to fluctuations in suicide ideation were also investigated (within subject design). A total of 214 participants were enrolled (61% women, age_mean_= 33 years, 45% presenting with suicide ideation during the course of the study). Greater frequency of vaping and using tobacco, opiates, and other substances, greater depression, anxiety, and PTSD symptoms, and greater COVID exposure and COVID-related worry were observed in individuals who presented with suicide ideation, compared to those that did not (p ≤ .0042). Increases in suicide ideation (within subject) was associated with increases in the frequency of alcohol and vaping, COVID-related worry, and PTSD symptoms (p ≤ .05). Gender-related differences in factors that relate to suicide ideation was observed. Within women, increases in frequency of alcohol use and PTSD symptoms and greater perceived early life trauma related to suicide ideation; while in men increases in vaping and COVID-related worry related to suicide ideation. This study further emphasizes the importance of investigating and identifying risk/resiliency factors for suicide-related thoughts and behavior in people who use drugs, including gender differences.

## Introduction

The COVID-19 pandemic has been associated with an increase in mental health problems that transcends diagnostic boundaries and age. While not all findings are consistent, an increase in depression, stress, anxiety, and alcohol/substance use in the general population has been observed since the initial lockdown in 2020^1–5^. Of particular concern is the rise in suicide-related thoughts and behavior^6,7^. While pandemic-related interventions, i.e. stay at home orders and physical distancing, is effective at lowering risk of disease transmission, these interventions may also contribute to economic burden and social isolation which raise risk for suicide^8^. Job loss and social isolation have both been linked to suicide risk^9^, with suicide risk evident even in communities with low COVID-19 prevalence^10^. Research is needed, especially longitudinal designs, to clarify risk factors for suicide-related thoughts and behavior to foster early identification and intervention^11^.

Pre-existing disorders are thought to increase risk for negative mental health outcomes, including suicide-related thoughts and behavior, due to COVID-19-related stressors^12^. These include, but are not limited to, post-traumatic stress disorder, pre-existing mood disorders, previous trauma exposure, and alcohol/substance use problems^13–18^. Indeed, prior work before the pandemic suggests mood disorders, trauma, alcohol and substance use disorders are risk factors for suicide-related thoughts and behavior^19–25^. How response to the pandemic interacts with these pre-existing disorders to contribute to suicide-related thoughts and behavior is still unclear. The majority of studies investigating risk factors for suicide-related thoughts and behavior, including emerging work investigating risk factors during the COVID-19 pandemic, have focused on group differences in the general population. These studies have not investigated risk factors that may be more predictive/specific to particular populations that are established to have a high risk for suicide, i.e., those with alcohol/substance use problems. The impact of the pandemic and factors that contribute to suicide-related thoughts and behavior may differ among high-risk groups—like those with alcohol/substance use problems—compared to the general population^26^. A greater understanding of factors that contribute to suicide-related thoughts and behaviors in substance users could foster novel interventions that are more specific and effective.

While variation in COVID distress and changes in mood and substance use behaviors are suggested to directly increase risk for suicide-related thoughts and behavior^9,13,27^, it is unclear if differences in these factors increase risk for suicide-related thoughts in individuals with alcohol/substance use problems or if risk differs by substance type or gender. Risk factors for, and maintenance of, alcohol/substance use disorders differ between males and females^28–32^. Studies also report females, compared to males, may be at greater risk for psychological distress, including depression and anxiety, during COVID-19^3,14^. As differences in risk for suicide-related thoughts and behavior—and relations between substance use during the pandemic and mental health outcomes—are suggested between males and females^33,34^, a greater understanding of the role(s) of gender is needed to inform prevention and early intervention strategies^35^.

This study investigates risk factors for suicide ideation during the COVID-19 pandemic in adults who use alcohol and other drugs. Participants completed up to three surveys over a six-month follow-up period. Data regarding COVID exposure, COVID-related worries, mood (including presence of suicide ideation), substance use, and trauma assessments were collected at each time point. At baseline, we also assessed for perceived early life trauma. We investigated factors that differed between individuals who reported suicide ideation over the follow-up period, compared to those that did not, and if risk factors differed between males and females (between subjects design investigating trait differences). Additionally, within those that reported suicide ideation at some point during the follow-up period we explored factors that related to fluctuations in severity of suicide ideation (a within subject design investigating state-dependent differences) and gender differences in factors associated with suicide ideation severity. We hypothesized the group that endorsed suicide ideation over the follow-up period would show greater perceived early life stress, COVID exposure and worries, trauma and mood symptoms, and frequency of alcohol/substance use compared to those that did not endorse suicide ideation during the study. We hypothesized females, compared to males, with suicide ideation would endorse greater trauma and internalizing symptoms, based on prior work suggesting variation in these factors may contribute to suicide risk in females^36,37^ and females may be at greater risk for internalizing symptoms in response to the pandemic^38^. We also hypothesized gender-differences in the relations between substance use and suicide risk would be observed based on recent work suggesting gender differences in the use of substances as a coping strategy during the COVID-19 pandemic^39^. We hypothesized these factors would predict fluctuations in suicide severity within individuals that reported suicide ideation over the six-month follow-up period.

## Methods

This study was designed to investigate patterns of substance use among people who use drugs during the COVID-19 pandemic. Participants were recruited through community-based harm reduction organizations across Austin, El Paso, and San Antonio, Texas, and social media and Craigslist advertisements. Eligibility criteria were: 1) 18 years or older; 2) resident of Texas; 3) self-reported use of illicit drugs at least once in the previous month (excluding marijuana use only) and 4) ability to read and speak English.

### Participants

A total of 214 participants were enrolled into the study (61% women, on average 33 years of age at enrollment) and completed three online surveys. Three-month follow-up surveys were completed by 188 participants (88% of sample) and 179 participants (84% of sample) completed six-month follow-up surveys. Across all participants and time points, a total of 572 participant surveys were completed for an average of 2.7 surveys per participant. To measure suicide ideation, at each time point participants were asked on a 5-point scale how much they had been distressed or bothered by thoughts of ending their life during the past two weeks (1=not at all; 2=a little bit; 3=moderately; 4=quite a bit; 5=extremely). Individuals were stratified into two groups: those who reported being distressed or bothered by thoughts of ending their life at any point during the six-month follow-up period (endorsing a 2 or higher at any point of assessment during the follow-up period), compared to those that did not report suicide ideation at any point during follow-up (endorsing a 1 at each assessment).

### Quantitative Data Collection

#### Measurements

##### Demographics Characteristics

Twenty-one questions regarding the participants’ sociodemographic characteristics were created by the authors and collected at the baseline survey. Questions included, but were not limited to, age, gender, race/ethnicity, and sexual orientation.

##### COVID-Related Mental and Behavioral Health

Data regarding COVID-related health impacts were captured using the CoRonavIruS Health and Impact Survey [CRISIS; www.crisissurvey.org;^40^].

###### COVID-19 Exposure/impact Status (Past Two Weeks):

Participants were asked to report possible exposure to Coronavirus/COVID-19, possible symptoms of COVID-19, family member diagnosis of COVID-19, and whether there had been any impacts on family members such as hospitalization, quarantine, and job loss because of COVID-19 during the past two weeks.

###### COVID Worries (Past Two Weeks):

Participants reported on a 5-point Likert scale (ranging from 1=not worried at all to 8=extremely worried) how worried they have been during the past two weeks about personal infection, friends and family being infected, and possible impacts on physical and mental health, as well as time spent reading or talking about COVID-19, and hope that the pandemic will end soon.

###### Substance Use (Past Two Weeks):

Participants reported how frequently they engaged in using alcohol, tobacco, vaping, opiates, marijuana, and other drug use (cocaine, crack, amphetamine, methamphetamine, hallucinogens, or ecstasy). The survey had eight frequency options ranging from 1=not at all to 8=more than once a day.

##### DSHS data on COVID fatalities per day

The number of COVID-19 deaths per day for the state of Texas was obtained from the Texas Department of State Health Services (DSHS) website^41^. This data was pulled in the form of .csv documents and then cross-referenced to match the respective day of survey completion for each participant, across the longitudinal study.

##### Mood and Trauma Assessments

###### Depression

The Center for Epidemiologic Studies Depression Scale (CES-D^42^) is a 20-item self-report instrument with high internal consistency (α=0.85) that measures depression symptoms such as feeling lonely, loss of appetite, and crying. Participants reported on how often they experienced symptoms on a 4-point Likert scale (0=rarely or none of the time/less than 1 day to 3=most or all of the time/5–7 days). Total scores range from 0 to 60 and scores of 16 or higher typically indicate risk for clinical depression^43^.

###### Anxiety

The Generalized Anxiety Disorder (GAD-7^44^) questionnaire is a seven-item, self-report instrument designed to measure symptoms of anxiety such as nervousness, feeling edgy, and worrying during the past two weeks. On a scale from 0=not at all to 3=nearly every day, participants rated their frequency of anxiety symptoms. Total scores range from 0 to 21. Cut-off point scores for mild, moderate, and severe anxiety are 5, 10, and 15 respectively. The GAD-7 has high internal consistency (α=0.92).

###### Posttraumatic Stress Disorder

The Primary Care PTSD Screen for DSM-5 (PC-PTSD-5^45^) is a five-item instrument with excellent diagnostic accuracy (Area Under the Curve/AUC=0.94). Participants are asked to respond yes (=1) or no (=0) to experiences of PTSD symptoms such as having nightmares, being on guard, and feeling numb or detached from people. Total scores range from 0 to 5 with a cut-off point score of 3 indicating probable PTSD diagnosis. Internal consistency is incalculable due to the binary nature of the response options.

###### Perceived Childhood Trauma

The Childhood Traumatic Events Scale (CTES^46^) was used to measure perceived childhood trauma. It has six options for trauma: “death of a very close friend or family member,” “major upheaval between your parents,” “traumatic sexual experience,” “victim of violence,” “extreme illness or injury,” and “other major upheaval.” Participants are instructed to 1) select the types of traumas they experienced before the age of 17; 2) indicate their age at the time of occurrence; 3) rate the severity of the trauma on a 7-point Likert scale (range from 1=not at all traumatic to 7=extremely traumatic); 4) rate the degree of confiding to others at the time on a 7-point Likert scale (range from 1=not at all to 7=confided a great deal). Internal consistency is incalculable due to the binary nature of the response options, but the scale is sensitive to clinical symptoms of early life trauma^47^.

#### Statistical Approach

##### Demographics

Differences in age, sex, race/ethnicity, and sexual orientation between individuals who presented with suicide ideation at any point during the follow-up period and individuals who never presented with suicide ideation during follow-up were investigated with student t-test or chi-squared test as appropriate.

##### Between Group Differences: Individuals Reporting Suicide Ideation (Over Course of 6 Month Follow-up) compared to Individuals who did not Report Suicide Ideation Over Follow-up

Mixed models were used to model main effects of sex, group (presenting with suicide ideation at some point vs. not endorsing suicide ideation at any point during study assessments), and sex by group interactions, with age included as a covariate and time of survey (baseline, three-month, six-month follow-up) as a within subject variable. Dependent variables investigated include prior 2-week substance use (including alcohol, vaping, tobacco, marijuana, opiates, and other substances), depression, anxiety, and PTSD symptoms, and perceived severity of childhood trauma. Following a significant group by sex interaction, models were stratified by sex and repeated. Following no significant group by sex interactions, the interaction term was dropped from the model and main effects of group or sex investigated. A parallel model was ran investigating between group differences in COVID worries and COVID exposure/impact, but additionally controlling for number of fatalities that occurred in Texas the day the CRISIS survey was filled out by a respective participant. Findings were considered significant at p≤.0042. (Bonferroni correction for 12 dependent variables being investigated).

##### Predictors of Fluctuations in Suicide Ideation in Individuals who Report Varying Levels of Suicide Ideation over Follow-up

Variables that significantly differed between individuals with suicide ideation over follow-up, compared to those without, were further investigated to see if fluctuations in these variables relate to fluctuations in suicide ideation over the follow-up period. This analysis was restricted to n=86 individuals in the group that showed suicide ideation over follow-up and had more than one survey completed with different levels of ideation reported across surveys (e.g., no ideation at baseline and ideation at a different time point or an individual that rated ideation as a 5 at one time point and ideation as a 3 at another). On average, each of these 86 participants completed 2.8 surveys providing 244 surveys included in this analysis. Age, sex, prior two-week alcohol, tobacco, vaping, opiate, other substance use, CRISIS worry, COVID exposure/impact, number of fatalities on the day the survey was completed, and PTSD symptoms were included in the model with time of survey (baseline, three-month, six-month follow-up) as a within subject variable, and suicide ideation score as a continuous dependent variable. Suicide ideation severity score was not normally distributed and was log transformed. Depression and anxiety symptom scores were not included in this model as these two variables highly correlated with PTSD symptom scores. We prioritized PTSD symptoms in these models since we observed a sex by suicide ideation group interaction on PTSD symptoms and investigating sex differences is a primary aim of this study. This model was repeated stratified by sex to explore for sex differences in risk factors contributing to fluctuation in suicide ideation. For this exploratory analysis, findings were considered significant at p≤.05.

## Results

### Demographics

Within this cohort, 45% (n = 97) reported suicide ideation at one point over the six-month follow-up period. There was no difference in age, sex, race/ethnicity, or sexual orientation between individuals who reported suicided ideation compared to those that did not (see Table 1).

#### Between Group Differences: Individuals Reporting Suicide Ideation (Over Course of 6 Month Follow-up) compared to Individuals who did not Report Suicide Ideation Over Follow-up

A main effect of group was observed for past month vaping (F = 8.5, p = .004), tobacco use (F = 13.4, p = .0003), opiates (F = 33, p < .0001), alcohol use (F = 8.5, p = .004), and other substances (F = 16.2, p < .0001). Specifically, individuals who reported suicide ideation at some point during the 6-month follow-up, compared to those that did not report suicide ideation, reported vaping and using tobacco, opiates, and other substances more, but reported drinking less. Individuals that reported suicide ideation had greater depression, anxiety, and PTSD symptoms (main effect of group, all p’s < .0001). There was a main effect of group on COVID exposure/impact (F = 16.2, p < .0001) and COVID worry (F = 14, p = .0002) with individuals who present with suicide ideation, compared to those that do not, reporting more COVID exposure/impact and more COVID worry. See table 2 for mean and standard deviation of dependent variables stratified by subgroup and gender and statistical comparison results.

There was a significant group by sex interaction (F = 11.2, p = .0009) on perceived severity of childhood trauma experienced, with women who reported suicide ideation, compared to those that did not, reporting greater perceived trauma (F = 51.3, p < .0001). There were no between group differences in perceived trauma in men reporting suicide ideation compared to men who did not report suicide ideation over follow-up (F = 2.4, p = .12). A main effect of sex was observed, with women presenting with greater depression symptoms, anxiety, and PTSD symptoms (all p’s ≤ .0005) compared to men. There was also a main effect of sex (F = 10.3, p = .001) on COVID worry, with women reporting more COVID-related worries.

#### Predictors of Fluctuations in Suicide Ideation in Individuals who Report Varying Levels of Suicide Ideation over Follow-up

Greater severity of suicide ideation was associated with greater recent alcohol use (F = 4.4, p = .04) and vaping (F = 6.7, p = .01) at the time of assessment. Greater severity of ideation also related to greater COVID-related worry (F = 8.6, p = .004) and PTSD symptoms (F = 5.4, p = .02) over the prior two weeks. When stratifying by gender, within men, greater severity of suicide ideation was associated with greater vaping (F = 4.7, p = .03) and COVID-related worry (F = 6.2, p = .02). Within women, greater recent alcohol use (F = 4.0, p = .05) and greater PTSD symptoms (F = 5.0, p = .03) was associated with greater suicide ideation.

## Discussion

The COVID-19 pandemic is resulting in a long-lasting mental health crisis. This includes increased risk for suicide-related thoughts and behavior following pandemic disruptions that span social, emotional, economic, and physical domains. Some individuals may be at greater risk for suicide-related thoughts and behavior, e.g., people who use drugs. Prevalence of suicide ideation was high in this study, with 45% of individuals reporting thoughts of suicide at least once during the six-month follow-up period. These estimates are higher than what has been reported in studies in the general population (6.2% prevalence^16^) and in other psychiatric, as well as non-psychiatric, populations considered at risk for suicide-related thoughts and behaviors^48–50^. As hypothesized, greater substance use (i.e., greater vaping and using tobacco, opiates, and other substances) was observed in individuals who presented with suicide ideation at some point over the follow-up period as well as greater depression, anxiety, and PTSD symptoms. Greater COVID exposure and COVID-related worry was also reported in individuals who presented with suicide ideation at some point during the follow-up period. The within subject longitudinal design (i.e., investigating symptom and behavioral correlates of fluctuations in severity of suicide ideation *in* individuals who presented with suicide ideation over the course of follow-up) support increases in suicide ideation is associated with increases in the frequency of substance use (i.e., alcohol and vaping), COVID-related worry, and PTSD symptoms. Results suggest these factors should be monitored in individuals who use substances and are at risk for suicide-related thoughts and behavior assessed when changes in these factors begin to emerge.

Findings also support our hypothesis that risk factors may differ by gender. Increases in frequency of alcohol use and PTSD symptoms related to increases in suicide ideation in women, but not men. Additionally, when comparing trait differences between groups we observed women who reported suicide ideation over follow-up reported greater perceived early life trauma, compared to women who did not present with suicide ideation. Greater early life trauma was not observed in men presenting with suicide ideation, compared to men who did not present with suicide ideation. This finding is supported by prior work suggesting women may be more at risk for PTSD symptoms and stress-sensitization may be more robust in women^51^, including fewer stressful events able to trigger psychiatric symptoms in women, compared to men following childhood adversity^52^. Additionally, PTSD is suggested to relate to greater risk for suicide-related thoughts and behavior in women, compared to men^36^. Gender differences in the relationship between alcohol use and suicide risk are also reported^20^. Additionally, an additive effect of early life stress and PTSD on risk for alcohol use disorders is reported in women^53^. In concert with these other studies, our findings support early life stress, PTSD symptoms, and alcohol use need to be closely monitored in people who use drugs, especially in women, with suicide risk assessment triggered if changes emerge.

Within men, but not women, increases in vaping and COVID-related worry related to increases in suicide ideation. Research on vaping is beginning to converge to support the relation between e-cigarette use and increased risk for suicide-related thoughts and behavior^54–58^. While few studies have investigated gender differences in this relationship, gender-differences in the pathways to e-cigarette use^59^ in addition to suicide-related thoughts and behavior^33^ are suggested. One prior study observed vaping in female adolescents is associated with greater risk for suicide ideation compared to male adolescents^54^. We observed increases in frequency of vaping relates to increases in suicide ideation in males, but not females. Discrepancy between findings suggesting gender differences between vaping and risk for suicide ideation could relate to age range being investigated (adult vs. adolescent), population being study (people who use drugs vs. general population), and/or differences in paradigms (e.g., longitudinal within subject design vs. cross-sectional). However, these studies converge to suggest gender differences may contribute to suicide thoughts and behavior in males and females who vape. Interestingly, there is emerging evidence suggesting that worry (excessive and unrealistic thoughts about future negative events) may contribute to e-cigarette use perception and barriers for quitting in men, but not women^60^. In light of our finding that increases in COVID-related worry also related to increases in suicide ideation in men, more research is needed on the interactions between psychosocial effects and vaping, including gender differences and underlying mechanisms, that may contribute to increased suicide-related thoughts and behavior.

While the within-subject longitudinal design of this study has many strengths, several limitations should be noted. Our sample was predominantly white; racial and ethnic minorities are disproportionately affected by the pandemic^61^. We cannot rule out confounding bias in the present study. For example, individuals who use substances more may be experiencing greater emotional turmoil and/or physical pain. We cannot infer causality. Individuals experiencing greater suicide-related thoughts and behavior may use substances as a means to cope with negative affect^62–64^. Future research is needed to investigate reasons for drug use as drug use motives may differentially relate to risk for detrimental outcomes^39^. Additionally, we assessed drug use frequency but did not assess quantity of drug use. We did not assess for diagnosis of mental health condition, including alcohol/substance use disorders, mood/anxiety disorders, or PTSD. More longitudinal studies are needed, with more frequent assessments, including quantity of drug use and clinical assessments to identify those with a history or current mental health condition, to better understand risk/resiliency factors that relate to emergence of suicide-related thoughts and behaviors, the temporal relations between factors, and commonalities/distinctions in risk/resiliency factors across vulnerable populations.

This study further emphasizes the importance of investigating and identifying risk/resiliency factors for suicide-related thoughts and behavior in people who use drugs, including gender differences. Future studies should investigate mechanisms that may contribute to stress sensitization and interactions with alcohol^65^, relations between psychosocial factors (i.e., feelings of worry) and vaping, and interactions with gender to identify novel targets for interventions and biobehavioral markers that may signal changes in risk for suicide-related thoughts and behaviors that are informed by gender. While some studies have found a decrease in substance use and suicidality in some during the pandemic^66^, it is important to remember that this may not generalize to all populations and risk may change over time^12^. Continued effort is needed to understand the long-term consequences of the pandemic, including risk for suicide-related thoughts and behavior^67^ in substance using populations^26^.

## Figures and Tables

**Table 1:** Demographic Characteristics

		NO SUICIDE IDEATION N = 117	SUICIDE IDEATION N = 97	*p value*
Number Female (%)		74 (63)	56 (58)	0.41
Mean Age (SD; Range)		33.2 (13.3; 18–67)	33.6 (11.3; 19–74)	0.81
Race/Ethnicity	Non-hispanic White (%)	49 (42)	45 (46)	0.32
	Hispanic (%)	36 (30)	32 (33)	
	African American (%)	13 (11)	4 (4)	
	Asian (%)	14 (12)	7 (7)	
	More Than One Race (%)	3 (3)	6 (6)	
	American Indian/Alaskan Native (%)	1 (1)	1 (1)	
	Prefer Not to Answer (%)	1 (1)	2 (2)	
Sexual Orientation	Gay/Lesbian	11 (9)	13 (13)	0.34
	Straight/Heterosexual	90 (77)	64 (66)	
	Bisexual/Pansexual	15 (13)	18 (19)	
	Other	1 (1)	2 (2)	

Demographic characteristics for individuals who presented with suicide ideation during the course of the study, compared to individuals who did not present with suicide ideation during the course of the study.
